# The influence of container geometry and thermal conductivity on evaporation of water at low pressures

**DOI:** 10.1038/s41598-018-33333-x

**Published:** 2018-10-11

**Authors:** Mohammad Amin Kazemi, Janet A. W. Elliott, David S. Nobes

**Affiliations:** 1 Department of Chemical and Materials Engineering, University of Alberta, T6G 1H9 Alberta, Canada; 2 Department of Mechanical Engineering, University of Alberta, T6G 1H9 Alberta, Canada

## Abstract

Evaporation is a ubiquitous phenomenon that occurs ceaselessly in nature to maintain life on earth. Given its importance in many scientific and industrial fields, extensive experimental and theoretical studies have explored evaporation phenomena. The physics of the bulk fluid is generally well understood. However, the near-interface region has many unknowns, including the presence and characteristics of the thin surface-tension-driven interface flow, and the role and relative importance of thermodynamics, fluid mechanics and heat transfer in evaporation at the surface. Herein, we report a theoretical study on water evaporation at reduced pressures from four different geometries using a validated numerical model. This study reveals the profound role of heat transfer, not previously recognized. It also provides new insight into when a thermocapillary flow develops during water evaporation, and how the themocapillary flow interacts with the buoyancy flow. This results in a clearer picture for researchers undertaking fundamental studies on evaporation and developing new applications.

## Introduction

A large amount of water evaporates daily from oceans, lakes, and rivers to keep the hydrologic cycle active. Sweat evaporates from our skin’s surface to regulate the temperature of our bodies. In the scientific disciplines of biology, agriculture, astronomy, medicine, and engineering, evaporation is a fundamental process to both academic and practical problems. Advances in understanding and modeling of evaporation have resulted in practical engineering applications such as spray cooling^1^, inkjet printing^2^, self-assembly of colloidal particles^3^, and DNA chip manufacturing^4^, to mention a few. Because of the importance of evaporation, publications in the area have increased significantly over the last 50 years^5^. Simultaneously, the application of evaporation is expanding with new discoveries such as exploiting the natural evaporation of water to generate electricity^6,7^ and mechanical work^8,9^, two promising advances towards clean energy technologies.

Studies on the evaporation of liquids in the past have indicated that evaporation is not as simple as it may seem at first glance. In fact, it is a complex phenomenon in which thermodynamics, hydrodynamics, heat transfer, and interfacial phenomena are inextricably interwoven. Most of the previous studies in this area have focused on evaporation of a liquid, mainly water, from a sessile drop into the air at atmospheric conditions. The reason for choosing such a configuration is partly to limit mathematical complexities present in asymmetric geometries given that a drop normally retains circular symmetry after being placed on a substrate. The evaporation of water into open air is believed to be governed by the diffusion of vapor molecules through the air^10^. In this limit, energy transport to the interface as well as the transport of molecules across the interface are assumed to occur much faster than the diffusive transport of molecules in the gas phase. A seminal study in this limit is the one conducted by Deegan *et al*.^11^ in which they described the coffee ring phenomenon, *i*.*e*., a ring-shaped solid residue left on the substrate after a drop of coffee is dried, during the evaporation of a sessile drop of suspension. They attributed this phenomenon to capillary flow from the center of the drop towards the periphery, induced by the necessity to replenish the intense evaporation loss at the drop periphery^12^. Inspired by this observation, they proposed an analytical function which expresses the distribution of evaporation flux on the drop surface as a function of drop contact angle. Later, Hu and Larson^13^ modified this function and derived a simpler expression which has been widely used in many theoretical studies so far. The essence of these expressions is that the evaporation is solely controlled by diffusive transport of molecules in the gas phase and that the evaporative cooling of the drop is negligible. However, experiments^14–18^ have shown that the evaporation from water drops resting on substrates with low thermal conductivity is remarkably slower than that from drops placed on high thermal conductivity materials, suggesting that heat transfer should also be taken into account. As a result, Sefiane and Bennacer^19^ and later Xu and Ma^20^ generalized the existing expressions by incorporating the cooling effects and separately proposed two more general expressions that could be used within a wider limit. Although the vapor diffusion-based theories of evaporation are commonly accepted and have been widely used, their accuracies and reliabilities are still open to question. For instance, the transport of molecules in the gas during evaporation of a drop may not be purely diffusive, the basic assumption of the current theories. Advection, induced by buoyancy effects, may be significant or even dominant, as highlighted by experiments^21,22^.

Despite the considerable advances in understanding the underlying physics involved in evaporation at atmospheric pressures^5,10,23^, evaporation at reduced pressures has not received much attention in the literature. Presumably, the reluctance to investigate this regime of evaporation stems from the fact that it is rare in nature. In addition, low-pressure experimental setups are intricate and the experimental procedures are rather complex. However, this regime of evaporation is not only of great importance in some current applications such as compact thermal technologies^24^, but also can direct us towards gaining a fundamental understanding of evaporation phenomena in general. This is because at reduced pressures, the well-known phenomenon of diffusion in the vapor no longer rules^10^, and other mechanisms are allowed to come into play. Given that the controlling mechanisms are different from that of evaporation at atmospheric pressure, different features in the fluids and at the phase boundary should be expected to occur. This points to a rich potential of low-pressure evaporation phenomena to emerge in new applications or to improve those which are currently controlled by evaporation at atmospheric pressures.

Reviewing the literature, one does not find many studies on this specific case of evaporation. Serious attempts to investigate evaporation at low pressures began in 1999 after the experimental measurements of temperature jumps at the liquid–vapor interface by Fang and Ward^25^. By using a fine thermocouple, they showed that there was a large temperature jump at the interface which was not detected before. The temperature jumps were larger at lower pressures and were strongly dependent on the heat flux from the vapor side^26,27^. The measured temperature jumps were inconsistent with the prevailing kinetic theory of gases that would predict a 10–20 times smaller temperature jump under the same condition^27,28^. To tackle such a discrepancy, statistical rate theory (SRT) of evaporation was applied^29,30^. SRT was consistent with the experimental temperature jumps in such a way that it could always predict the pressure of the vapor phase within the experimental uncertainty without using any fitting parameter. Several studies^31–39^ were conducted afterward supporting the reliability of SRT in describing the evaporation flux from the interface. Some studies highlighted the remarkable contribution of thermocapillary flow, present within the first 500 µm of the liquid, to evaporation by assessment of the energy balance at the interface^33,34,40,41^. These studies however, did not provide a detailed explanation of when the thermocapillary flow exists and why it was absent in some experiments under the same thermodynamic conditions^36^. While most of the studies have revolved around the assessment of SRT by exploring transport phenomena through measuring temperature gradients in the vicinity of the interface, the role of instabilities in the bulk liquid has not yet been identified clearly. In almost all of the past studies, it was assumed that the effects of buoyancy in the liquid were eliminated during the evaporation of water if the temperature at the bottom of the container was kept at 4 °C where water would be at its highest density. However, experimental observations of the convection pattern below an evaporating meniscus^42,43^ as well as numerical simulations^43^ suggest that buoyancy effects are dominant in the bulk liquid even though the bottom temperature is maintained at 4 °C.

Accordingly, this paper investigates how the bulk flow of the liquid, heat transfer, geometric configuration and thermal properties of the evaporation vessel determine the evaporation from the interface. To elucidate how the flow instabilities influence the energy transport to the interface, we have developed a mathematical model and simulated the low-pressure evaporation process of water within the four different geometric configurations for which experimental results have been reported in the literature. The model takes into account the hydrodynamics of the liquid and vapor using the Navier–Stokes equations, conduction and convection equations in the bulk of fluids, and conduction through the solid walls of the liquid container. All the physical properties of the fluids are assumed to change with temperature. At the liquid–vapor interface, the evaporative cooling effects due to the phase change and thermocapillary convection due to the variation of surface tension with temperature are considered. The simulated evaporation fluxes were calculated by averaging the local evaporation fluxes expressed by either SRT or the energy balance equation (both give the same value of evaporation flux). The strongly coupled system of equations was discretized using finite element based software (COMSOL Multiphysics, COMSOL Inc.) and the velocity and temperature fields within the fluids, as well as the evaporation flux, were calculated. The reliability of the model in predicting the evaporation phenomenon was confirmed against earlier experimental studies for two container geometries, namely a cylindrical tube^43^ and a rectangular cuvette^44^. In the study reported herein, we applied the model to two more container geometries existing in the literature for which the velocity field data has not been measured experimentally. By comparing the simulation results across the four different geometries, we explore how the thermal boundary conditions that are dictated by the geometry as well as the thermal conductivity of the evaporation cell strongly affect the flow field in the liquid, which in turn impacts the evaporation from the interface.

## Results

We have previously developed a mathematical model of the evaporation process at low pressure (see refs^43,44^) to describe the velocity, pressure, and temperature distributions in the fluids, as well as the temperature distribution in the solids, and finally the evaporation flux. For evaporation of water within a cylindrical tube (borosilicate glass)^43^ and a rectangular cuvette (quartz)^44^, we previously experimentally measured the velocity field in the liquid below the interface experimentally using particle image velocimetry^44–47^. We assessed the validity of the model by comparing the model predictions of the velocities in the liquid, temperatures of the liquid and vapor along the vertical centerline, and global evaporation fluxes with the experimental data^43,44^. The model showed excellent agreement with the temperatures measured in the liquid and vapor by a thermocouple, and could predict the two dimensional velocity field in the center of the cylindrical tube and the three dimensional velocity field in the volume of the rectangular cuvette very well^43,44^. In the study reported herein, the model was applied to two other geometries existing in the literature^27,33^ although experimental velocity data are not available for either. We should note that the model used no fitting parameters to predict the abovementioned quantities and only required the pressure of the vapor, the ambient and bottom container temperatures, and the magnitude of temperature jumps as input, which were all measured for each experiment. In the simulations, the values of temperature jumps were chosen from a fitted line to the available data at various pressures.

**Figure 1:**
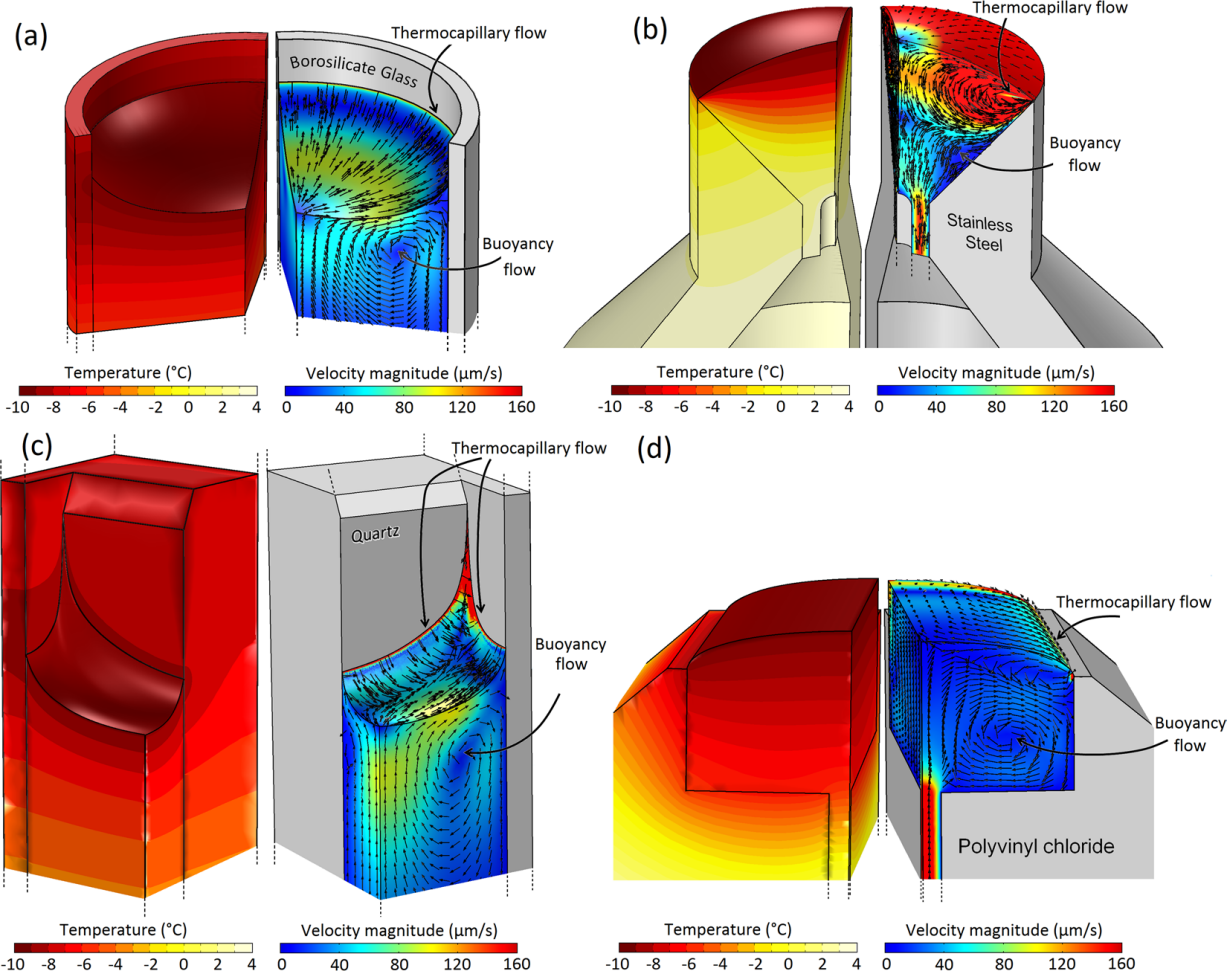
Simulated velocity and temperature distributions in water while it evaporates from different geometries at 300 Pa. Different panels show simulation results for the experimental geometries used in the studies of (**a**) Kazemi *et al*.^43^, (**b**) Ward and Duan^33^, (**c**) Kazemi *et al*.^44^, and (**d**) Badam *et al*.^27^. In each panel, the predicted temperature distribution in the liquid is shown on the left side and the predicted velocity magnitude as well as the normalized velocity arrows are shown on the right side. Only the upper parts of the actual experimental geometries (complete geometries shown in Fig. S1 in the Supplementary Material) are shown to focus on important parts of the flows. In the experiments, at positions below those shown in the figure, the temperatures of the bottom walls in (**a**) and (**c**) and of the entering liquid in (**b**,**d**) were all kept at 4 °C. The thermal conductivities of the solids are (**a**) *k*_*s*_ = 1.14 W/(m K), (**b**) *k*_*s*_ = 16 W/(m K), (**c**) *k*_*s*_ = 3 W/(m K), and (**d**) *k*_*s*_ = 0.13 W/(m K).

Figure 1 shows the simulated velocities and temperatures within the liquid as water evaporates in four different geometries at a typical pressure of 300 Pa. In the corresponding experiments, the temperatures at the bottom of the containers were maintained at 4 °C hypothesizing that this would eliminate buoyancy effects in the liquid. However, as the simulations suggest, the buoyancy effects play a significant role in the flow pattern of the liquid in the bulk and consequently the evaporation from the interface. As can be seen in Fig. 1, one may think that the clockwise circulation of the liquid in the bulk (in the right half-plane in Fig. 1) induced by the buoyancy effects should enhance the evaporation rates as it brings warm liquid from the bottom to the cold interface. However, by removing the buoyancy effects in the simulations (by setting gravitational acceleration *g* to 0 m/s^2^), we noticed that the evaporation rates in all geometrical configurations increase, suggesting that the buoyancy effects diminish the evaporation from the interfaces. In particular, the large-scale flow in the bulk of the liquid generated due to buoyancy resists the development of a thermocapillary flow at the interface (see Fig. S3 in the Supplementary Information) and reduces the evaporation rates accordingly. The direction of curvature of the interface is also a key factor in determining how the buoyancy and thermocapillary forces interact near the interface. That is to say, the isothermal surfaces near the interface in the liquid shown in Fig. 1 tend to follow the shape of the interface. As a result, for the two concave interfaces in Fig. 1(a,c), the resultant density distribution below the interface propels the liquid near the solid wall downward and consequently creates a large vortex (clockwise in the right half-plane) that opposes the thermocapillary flow. This prevents thermocapillary flow in (a) and (c) from spreading over the interface. In contrast, for the two convex interfaces in Fig. 1(b,d), the density variation below the interface is such that it creates a counterclockwise vortex (counterclockwise in the right half-plane) that assists the thermocapillary flow. This allows thermocapillary flow in (d) to easily spread over the entire interface although the container has a smaller thermal conductivity (*k*_s_ = 0.13 W/(m K)) compared to those of (a) and (c) (*k*_s_ = 1.14 W/(m K) and *k*_s_ = 3 W/(m K), respectively) in which thermocapillary flow does not occur. In Fig. 1(b) with both a large container thermal conductivity and a convex interface, a very strong thermocapillary vortex is evident (See Fig. S2 of the Supplementary Information).

**Figure 2:**
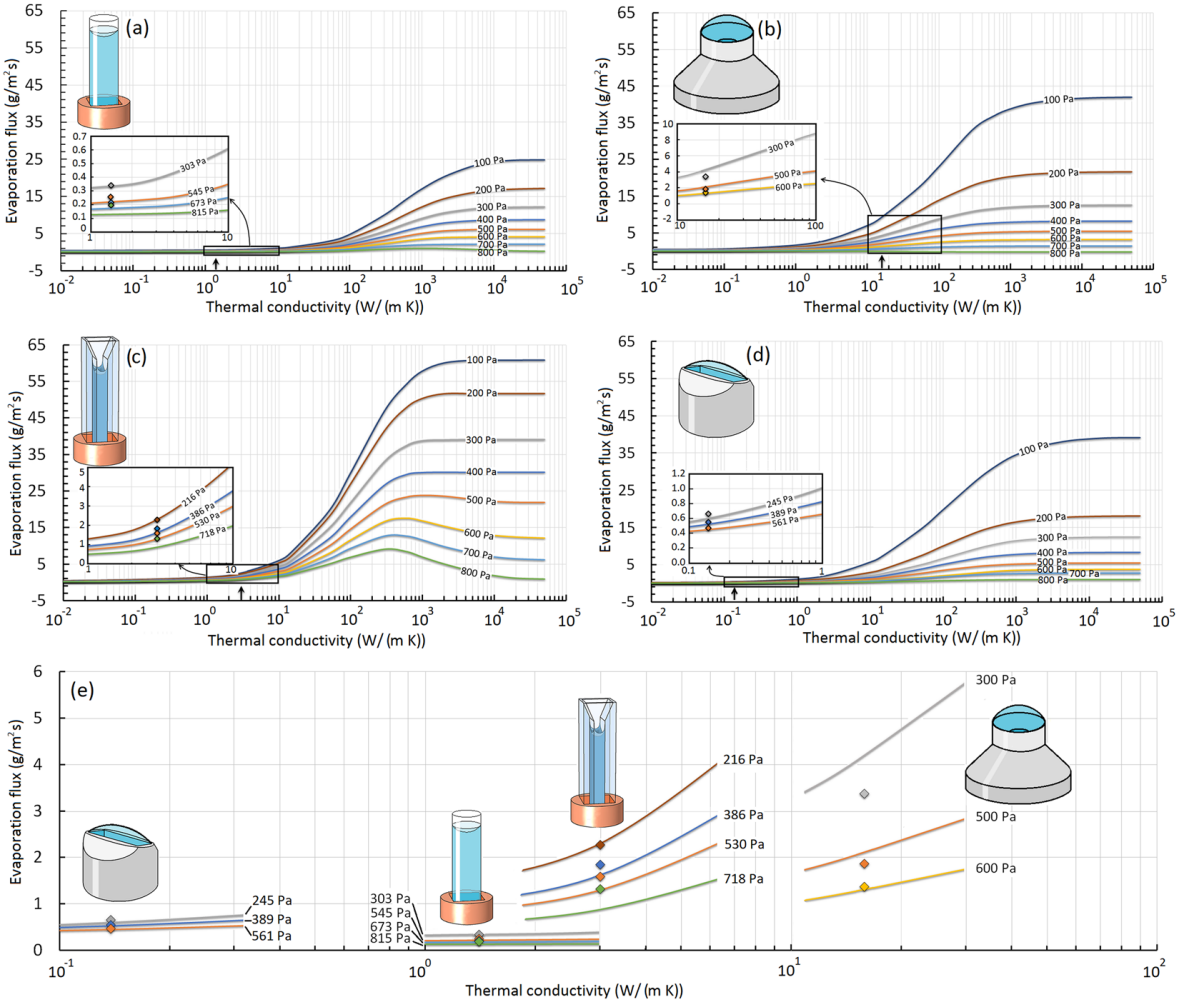
Variation of simulated water evaporation flux versus the thermal conductivity of the container for different geometries studied experimentally in the literature. The solid curves show the simulated fluxes at different pressures and the data points show the measured values at the corresponding pressures shown on the curves. Different panels show the simulation results and measured evaporation fluxes for the experimental geometries used in the studies of (**a**) Kazemi *et al*.^43^, (**b**) Ward and Duan^33^, (**c**) Kazemi *et al*.^44^, and (**d**) Badam *et al*.^27^. The arrows on the x-axes indicate the thermal conductivity of the container used in the experiments. Insets show expanded regions at the thermal conductivities of the experiments. Panel (**e**) shows a comparison between the evaporation fluxes in different geometries and how accurately the model can predict the evaporation fluxes across geometries and across container thermal conductivities.

In previous studies of evaporation of a sessile drop on a substrate at atmospheric pressures, the thermal conductivity of the substrate was shown to have a significant effect on circulation patterns inside the drop^48^ and subsequently on evaporation rates^14–18^. This means that the heat transfer mechanism plays a crucial role during evaporation at atmospheric pressures. In this study, to examine the effect of heat transfer on evaporation at low pressures, the thermal conductivities of materials in the simulations were purposely varied between 10^−2^ W/(m K) and 10^5^ W/(m K), and the evaporation fluxes in the four different geometries were calculated. Figure 2 (a–d) shows the variation of the simulated evaporation flux with container thermal conductivity within the reduced pressure range of 100 Pa to 800 Pa. The inset magnifications provide a comparison between the simulation predictions and the available experimental measurements taken from the relevant references. To understand results for different geometries with respect to one another, the inset regions are amalgamated and shown together in Fig. 2(e). This panel clearly highlights the universality of the numerical modeling approach in predicting the evaporation rates for both the trend in container thermal conductivity and the effect of the container geometry. At small values of solid thermal conductivity (*i*.*e*., *k*_*s*_ < 1) in Fig. 2(a–d), the evaporation rates are very small for all geometries and they slightly increase with thermal conductivity. However, at a certain thermal conductivity for each geometry, the evaporation flux exhibits a remarkable enhancement which corresponds to *k*_*s*_ = 1 W/(m K) for geometries (b), (c), and (d) and *k*_*s*_ = 10 W/(m K) for geometry (a). As *k*_*s*_ becomes larger, the evaporation flux increases until it approaches an asymptotic value at very high values of *k*_*s*_. This asymptotic value is different for different geometries and also depends on the pressure in the vapor phase for the same geometry. At this condition, the rectangular geometry (c) has the highest evaporation flux compared to the other three geometries due to the high evaporation from the thin liquid fingers formed at the corners as shown in Fig. 1(c). The results for the convex geometries in Fig. 2(b,d) show almost the same behavior at all pressures, while the evaporation rate from the cylindrical tube (a) is the lowest under this circumstance. Here, we should emphasize that the asymptotic value of the evaporation flux strongly depends on the temperature boundary condition. This means that larger asymptotic values of evaporation flux could be achieved if a temperature higher than 4 °C were imposed at the bottom of the container. This is because at very high thermal conductivities, the container acts as a thermal superconductor and the whole solid gains a uniform temperature equal to that imposed at the bottom boundary. According to the simulations, by increasing the thermal conductivity, the thermocapillary flow becomes stronger and tends to spread over the interface. It is interesting to note that the significant increase in the evaporation flux observed in Fig. 2(a–d) has nothing to do with the occurrence of a thermocapillary flow at the interface. To be more specific, consider the rectangular geometry in Figs 1(c) and 2(c) in which a thermocapillary flow does not occur at small values of *k*_*s*_. For this geometry, the thermocapillary forces become stronger upon increasing the thermal conductivity and compete with the large buoyancy vortex until a thermocapillary flow develops completely across the interface at *k*_*s*_ = 15 W/(m K). At this point, the evaporation flux is still much smaller than the asymptotic value and further increase of *k*_*s*_ is required to achieve the maximum evaporation rate. Other evidence demonstrating that there is no relation between the occurrence of a thermocapillary flow and the remarkable increase in the evaporation flux can be shown by geometry (a). For this geometry, due to the concave shape of the interface, a thermocapillary flow never spreads over the interface (see Fig. S2 in the Supplementary Information), even at the highest thermal conductivity and the lowest pressure. However, a significant increase in the evaporation rates can be seen after *k*_*s*_ = 10 W/(m K). Finally, for the convex geometries, (b) and (d), a thermocapillary flow always exists at the interface since the thermocapillary flow is in the same direction as the buoyancy vortex. However, the evaporation fluxes for these two geometries are still very small at small values of thermal conductivity even though a thermocapillary flow always exists for these convex geometries.

This study demonstrates several important insights that are directly in contrast to prevailing thought that was based on conclusions of authors that only studied a single geometry and a single container thermal conductivity. As demonstrated by the validated mathematical model, evaporation of water at reduced pressures strongly depends on the geometry, thermal properties of the container, and the curvature of the interface. The assumption that water is buoyancy stabilized if the bottom container temperature is set to 4 °C is incorrect. The bulk flow in the liquid, which has not received enough attention in past studies, can greatly affect the evaporation by either promoting or suppressing the thermocapillary flow at the interface. As a result, future studies on the topic should include all of these parameters to provide better insight into the complex problem of evaporation and to improve practical applications.

The results of the numerical simulations demonstrated here are also of great significance in developing theories of evaporation and condensation. As the results suggest, by using a high thermal conductivity material such as graphene (*k*_*s*_ = 5 × 10^4^ W/(m K), the heat transfer resistances are smaller and the evaporation fluxes are higher. Using the highest thermal conductivity possible would be more desirable for thermodynamicists to experimentally explore the accuracy of the existing theoretical expressions of evaporation and condensation. This is because the evaporation flux is at its maximum value at that specific condition while the heat transfer limitation is minimal. At this condition, the evaporation is closer to the state in which it would be controlled by the kinetic effects at the interface. Therefore, the conclusions are less prone to be affected by the errors associated with the measurements of the interfacial temperatures and pressures. This is the limit of evaporation in which assessment of the existing evaporation expressions such as SRT and KTG (kinetic theory of gases) in predicting some fundamental phenomena occurring at the interface such as temperature jumps and condensation coefficients can be better undertaken. If experiments are performed in the limit of evaporation that is mainly governed by heat transfer, as was the case in most of previous studies, the conclusions about these concepts may not be accurate since the interfacial phenomena have not played a role in evaporation from the interface. This is perhaps the reason that the SRT expression for evaporation flux agrees with a wide range of temperature jumps^28^, or that the evaporation coefficients obtained from the experiments are highly scattered between zero and one^49^.

## Methods

The velocity and pressure distributions in the liquid and vapor were approximated using the Navier–Stokes equations for steady and compressible flow of a Newtonian fluid. Heat transfer in the fluids was assumed to take place by conduction and convection mechanisms and radiative heat transfer was ignored as discussed in the supporting information of ref.^28^. The heat fluxes were determined by Fourier’s law of conduction and the heat generated by viscous dissipation was neglected. The simulated evaporation fluxes were calculated by averaging the local evaporation fluxes expressed by either the SRT or the energy balance equation (both give the same value of evaporation flux). The physical properties of the fluids were assumed to vary with temperature and can be found in ref.^43^. The equations and boundary conditions used in the simulation are listed in Part I of the Supplementary Information.

To solve the system of equations the model was implemented in commercial finite element software (COMSOL Multiphysics® version 5.2a, COMSOL Inc.) and the equations were discretized using second order triangular elements for velocity components and linear triangular elements for the pressure. For each simulation, an extensive convergence study was carried out to verify that the solutions were independent of the mesh size^45^. Details of the simulation procedure can be found in refs^43–45^.

## Electronic supplementary material

Supplementary Information
