# Supplementary material for: The influence of container geometry and thermal conductivity on evaporation of water at low pressures
Source: Sci Rep. 2018 Oct 11;8:15121. doi: 10.1038/s41598-018-33333-x (PMC6181933; doi:10.1038/s41598-018-33333-x)
Supplement: Supplementary file 1 — Supplementary Information [file 41598_2018_33333_MOESM1_ESM.pdf]

Supplementary Information:

The influence of container geometry and thermal conductivity on evaporation of water at low pressures

Mohammad Amin Kazemi[1], Janet A.W. Elliott[1,*], David S. Nobes[2,*]

[1]Department of Chemical and Materials Engineering

[2]Department of Mechanical Engineering

University of Alberta, Canada, T6G 1H9

*Corresponding authors' email addresses: janet.elliott@ualberta.ca, dnobes@ualberta.ca

Part I – Description of the Numerical Approach

Mathematical Model

In this section, the equations and boundary conditions used in simulation of the evaporation phenomena in the four geometries shown in the main paper are briefly presented. All the equations are written for steady state conditions. More discussion on the selection of the equations is given in ref. 44. The complete geometries used in the simulations including the dimensions are illustrated in Figure S1.

In the bulk of the fluids, the continuity and momentum equations are:

$$\nabla \cdot \boldsymbol{u}^L = 0, \tag{S1}$$

$$\nabla \cdot (\rho^V \boldsymbol{u}^V) = 0, \tag{S2}$$

$$\rho^L(\boldsymbol{u}^L \cdot \nabla)\boldsymbol{u}^L = \nabla \cdot [-P^L\boldsymbol{I} + \mu^L(\nabla\boldsymbol{u}^L + (\nabla\boldsymbol{u}^L)^{tr})] + \rho^L\boldsymbol{g}, \tag{S3}$$

$$\rho^V(\boldsymbol{u}^V \cdot \nabla)\boldsymbol{u}^V = \nabla \cdot \left[-P^V\boldsymbol{I} + \mu^V(\nabla\boldsymbol{u}^V + (\nabla\boldsymbol{u}^V)^{tr}) - \frac{2}{3}\mu^V(\nabla \cdot \boldsymbol{u}^V)\boldsymbol{I}\right] + \rho^V\boldsymbol{g}, \tag{S4}$$

where $\boldsymbol{u}$ is the velocity vector, superscripts L and V refer to the liquid and vapor phases, ρ is the density, P is the pressure, $\boldsymbol{I}$ is the identity matrix, μ is the viscosity, and $\boldsymbol{g}$ is the gravitational acceleration vector. The superscript tr means transposed.

The temperatures of the liquid and vapor were determined from heat transfer convection–diffusion equations:

$$\rho^L C_p^L \boldsymbol{u}^L \cdot \nabla T^L = \nabla \cdot (k^L \nabla T^L), \tag{S5}$$

$$\rho^V C_p^V \boldsymbol{u}^V \cdot \nabla T^V = \nabla \cdot (k^V \nabla T^V), \tag{S6}$$

where k is the thermal conductivity and C_p is the specific heat capacity at constant pressure. In solids, the energy balance is simplified to:

$$\nabla \cdot (k^S \nabla T^S) = 0. \tag{S7}$$

where superscript S refers to the solid phase.

Boundary Conditions

The boundary conditions used to solve the above equations are listed in the following. At the liquid–vapor interface, the mass balance requires that:

$$m'' = \rho^L \boldsymbol{u}^L \cdot \boldsymbol{n} = \rho^V \boldsymbol{u}^V \cdot \boldsymbol{n}, \tag{S8}$$

where m'' is the net evaporation mass flux and $\boldsymbol{n}$ is the unit normal vector at the interface which points toward the vapor. The net evaporated mass flux across the liquid–vapor interface may be calculated by the statistical rate theory of interfacial transport (SRT) as[30]:

$$m'' = 2\left(\frac{P^{sat}\exp\left(V_\infty^L\left(P^L - P^{sat}\right)/\bar{R}T^L\right)}{\sqrt{2\pi\bar{R}T^L}}\right)\sinh(\Delta S/\bar{R}), \tag{S9}$$

where P^{sat} is the saturation pressure at T^L, V_∞^L is the specific volume of the saturated liquid, $\bar{R}$ is the individual gas constant, sinh denotes the hyperbolic sine function, and ΔS is the change in the entropy due to the phase change. ΔS can be calculated by[30]:

$$\frac{\Delta S}{\bar{R}} = \left\{4\left(1 - \frac{T^V}{T^L}\right) + \left(\frac{1}{T^V} - \frac{1}{T^L}\right)\sum_{i=1}^{3}\left(\frac{\Theta_i}{2} + \frac{\Theta_i}{\exp(\Theta_i/T^V) - 1}\right) + \frac{V_\infty^L}{\bar{R}T^L}\left(P^L - P^{sat}\right) + \ln\left[\left(\frac{T^V}{T^L}\right)^4 \frac{P^{sat}}{P^V}\right] + \ln\left[\frac{q_{vib}(T^V)}{q_{vib}(T^L)}\right]\right\}, \tag{S10}$$

where Θ_i is the ith vibrational frequency of the water molecules and q_{vib} denotes the vibrational partition function given by:

$$q_{vib}(T) = \prod_{i=1}^{3}\frac{\exp(-\hbar\Theta_i/2k_B T)}{1 - \exp(-\hbar\Theta_i/k_B T)}, \tag{S11}$$

The momentum balance at the liquid–vapor interface can be described by:

$$(\boldsymbol{\tau}'_L - \boldsymbol{\tau}'_V) \cdot \boldsymbol{n} = \sigma(\nabla_t \cdot \boldsymbol{n})\boldsymbol{n} - \nabla_t\sigma + m''(\boldsymbol{u}^L - \boldsymbol{u}^V), \tag{S12}$$

where $\boldsymbol{\tau}'$ is the total stress tensor, subscript t denotes the unit tangent vector at the interface, ∇_t is the surface gradient operator, $(\nabla_t \cdot \boldsymbol{n})$ is the curvature of the interface, and σ is the liquid surface tension which is assumed to be a function of temperature.The total stress tensors in liquid and vapor are defined as:

$$\boldsymbol{\tau}'_L = -P^L\boldsymbol{I} + \mu^L(\nabla\boldsymbol{u}^L + (\nabla\boldsymbol{u}^L)^{tr}), \tag{S13}$$

and

$$\boldsymbol{\tau}'_V = -P^V\boldsymbol{I} + \mu^V(\nabla\boldsymbol{u}^V + (\nabla\boldsymbol{u}^V)^{tr}) - \frac{2}{3}\mu^V(\nabla \cdot \boldsymbol{u}^V)\boldsymbol{I}. \tag{S14}$$

To determine the temperature distribution at the interface, the energy balance is written as:

$$(k^V\nabla T^V - k^L\nabla T^L) \cdot \boldsymbol{n} - (\boldsymbol{n} \cdot \boldsymbol{\tau}_l) \cdot \boldsymbol{u}^L + (\boldsymbol{n} \cdot \boldsymbol{\tau}_v) \cdot \boldsymbol{u}^V = m''h^{LV}, \tag{S15}$$

where $\boldsymbol{\tau} = \boldsymbol{\tau}' + P\boldsymbol{I}$ is the viscous stress tensor and h^{LV} is the enthalpy of vaporization which is taken to be a function of temperature. At the liquid–vapor interface, we assume a no-slip condition ($\boldsymbol{u}^L = \boldsymbol{u}^V$) as well as a temperature jump condition. The values of temperature jumps were chosen from a fitted line to the available data at various pressures. At the solid–liquid and solid–vapor boundaries, the temperatures of both phases as well as the heat fluxes on each side were assumed to be equal. Details of thermal boundary conditions can be found in Figure S1. The rate of the mass entering the liquid domain should be defined in such a way that it compensates for the mass lost at the interface, thus:

$$\dot{m}_{in} = \iint m''(x, y, z)dA_I, \tag{S16}$$

where $\dot{m}_{in}$ is the inlet mass flow rate and A_I is the area of the interface.

At the vapor outlet, the normal stress is specified as:

$$\boldsymbol{\tau}'_V \cdot \boldsymbol{n} = -P_0\boldsymbol{n}, \tag{S17}$$

where P_0 is the pressure measured by the pressure transducer. At the vapor outlet, it is also assumed that the temperature does not vary across the boundary ($\nabla T^V \cdot \boldsymbol{n} = 0$).

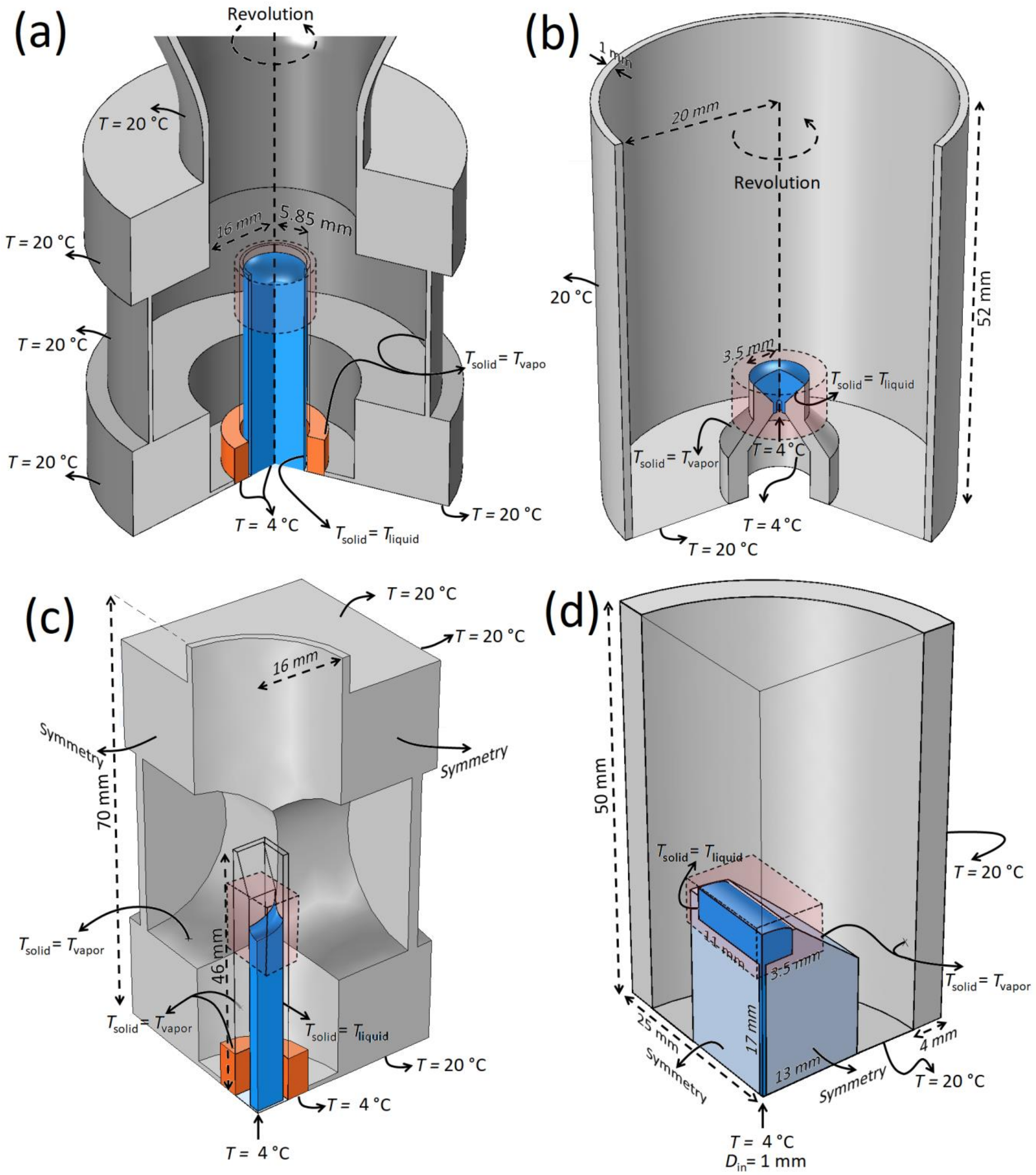

Figure S1. The configurations of the simulated geometries that represent the studies of (a) Kazemi *et al.*[43] (b) Ward and Duan[33] (c) Kazemi *et al.*[44] and (d) Badam *et al.*[27]. The upper parts of the vacuum chamber in (a) and (c) are not shown but can be found in the corresponding references. The vacuum chambers in (a), (b), and (c) were made up of stainless steel while in (d), the chamber was made up of Perspex. The thermal boundary conditions used in the simulations are annotated by black arrows. In each panel, the pale red area enclosed by black dashed lines highlights the region within which the simulated velocities and temperatures are illustrated in the main paper.

Part II – The flow field within the liquid phase

The following discussion is provided to support statements in the main paper. Specifically, the role of the thermal conductivity of the container holding the liquid water is described by exploring the velocity field that is generated within the fluid flow. The results presented here for a single operating pressure of P^V = 300 Pa are representative of all the numerical simulations described in the main paper. The range of values of thermal conductivity presentated here in the Supplementary Information have been chosen to fully demonstrate the impact of container thermal conductivity on the liquid flow field.

Figure S2 shows how the velocity field in the liquid changes by varying the thermal conductivity of the wall of the container holding the water for the four geometries described in the main paper. For evaporation from a cylindrical tube (Kazemi *et al.*[43]), geometry (a), increasing the thermal conductivity of the wall strengthens both the large buoyancy vortex and the thermocapillary flow near the three phase contact line. When k_s = 1 W/(m K), no thermocapillary vortex was observed in the simulation while for k_s = 10, 100, 1000 W/(m K), a small vortex appears near the contact line and becomes stronger with increasing thermal conductivity of the wall. For evaporation from an axisymmetric reversed-flow funnel (Ward and Duan[33]) geometry (b), a thermocapillary vortex always exists even at the lowest thermal conductivity of the wall. When k_s = 1 W/(m K), the presence of buoyancy driven vortices in the liquid is not evident. As the thermal conductivity increases to k_s = 10 W/(m K), a small buoyancy vortex appears in the liquid. This buoyancy vortex shrinks as the thermal conductivity increases further and completely disappears for k_s = 1000 W/(m K). The change in the liquid velocity field for geometry (c), a large aspect ratio rectangular cuvette (Kazemi *et al.*[44]), is similar to that for geometry (a), with the difference that when k_s = 1 W/(m K), no thermocapillary vortex was observed in the simulation. For evaporation from a long aspect ratio trough with continuous feed (Badam *et al.*[27]), geometry (d), at small values of thermal conductivity of the wall (k_s = 1 W/(m K)), both a buoyancy vortex and a thermocapillary vortex exist. However, for other larger values of thermal conductivity, the thermocapillary vortex becomes dominant and diminishes the buoyancy vortex in the liquid.

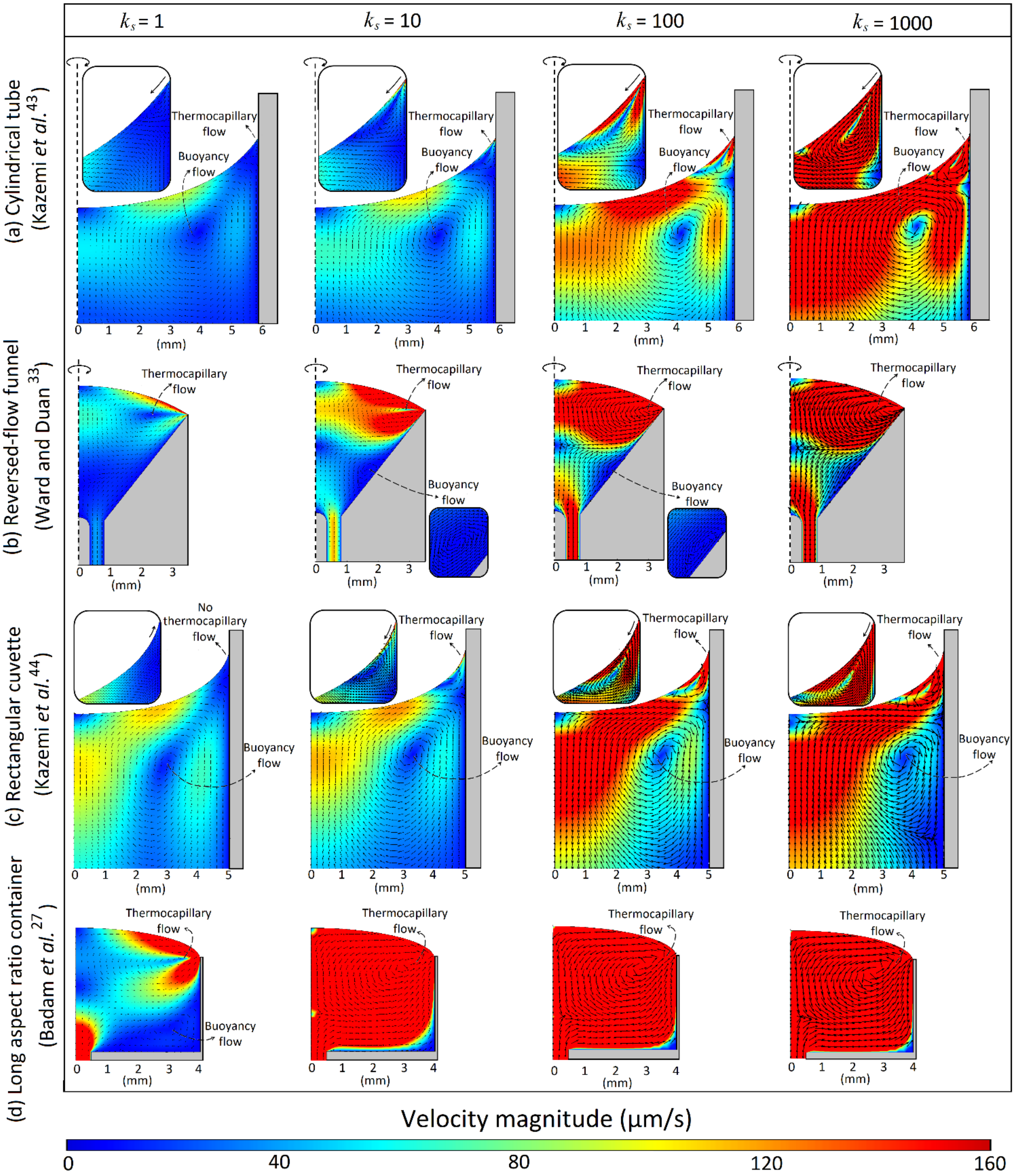

Figure S2. Interactions between the buoyancy and thermocapillary forces and the flow field that is generated during the evaporation of water in four geometries obtained from numerical simulations. Different panels show the velocity field simulation results for the experimental geometries used in the studies of (a) Kazemi *et al.*[43] (b) Ward and Duan[33] (c) Kazemi *et al.*[44] and (d) Badam *et al.*[27]. The colors in the background show the velocity magnitudes. The arrows represent the direction of the flow. In the simulations, the pressure in the vapor P^V is set to 300 Pa.

Figure S3 shows how the liquid flow changes when either buoyancy or thermocapillary effects are ignored in the simulation of evaporation from the axisymmetric reversed-flow funnel investigated by Ward and Duan[33]. In Figure S3 (a), the thermocapillary effects are eliminated by setting a constant value of 0.076 N/m for the surface tension in the simulation. As can be seen, in the absence of thermocapillary effects, two buoyancy driven vortices form in the liquid. The larger one rotates clockwise which is due to the effect of the warmer container wall. The smaller one rotates in the counter-clockwise direction near the axis of symmetry of the container and the liquid–vapor interface. This is due to the special shape of the density contours affected by the convex curvature of the interface. Figure S3 (b) shows the liquid flow pattern when the buoyancy effects are removed in the simulation by setting the gravitational acceleration g to 0 m/s^2. As can be seen in Figure S3 (b), in the absence of buoyancy effects, a large thermocapillary vortex which is rotating counter-clockwise develops over a significant portion of the liquid domain. In Figure S3 (c), both buoyancy and thermocapillary effects are activated in the simulation. Figure S3 (c) shows the competition between these two forces and the generated flow field. In the upper part of the liquid, the thermocapillary effects are dominant and a large vortex due to thermocapillary effects exist immediately below the interface. However, the velocity magnitude of this vortex is smaller than that shown in Figure S3 (b) since the large buoyancy vortex that would exist in the absence of thermocapillary effects (Figure S3 (a)) resists the thermocapillary forces.

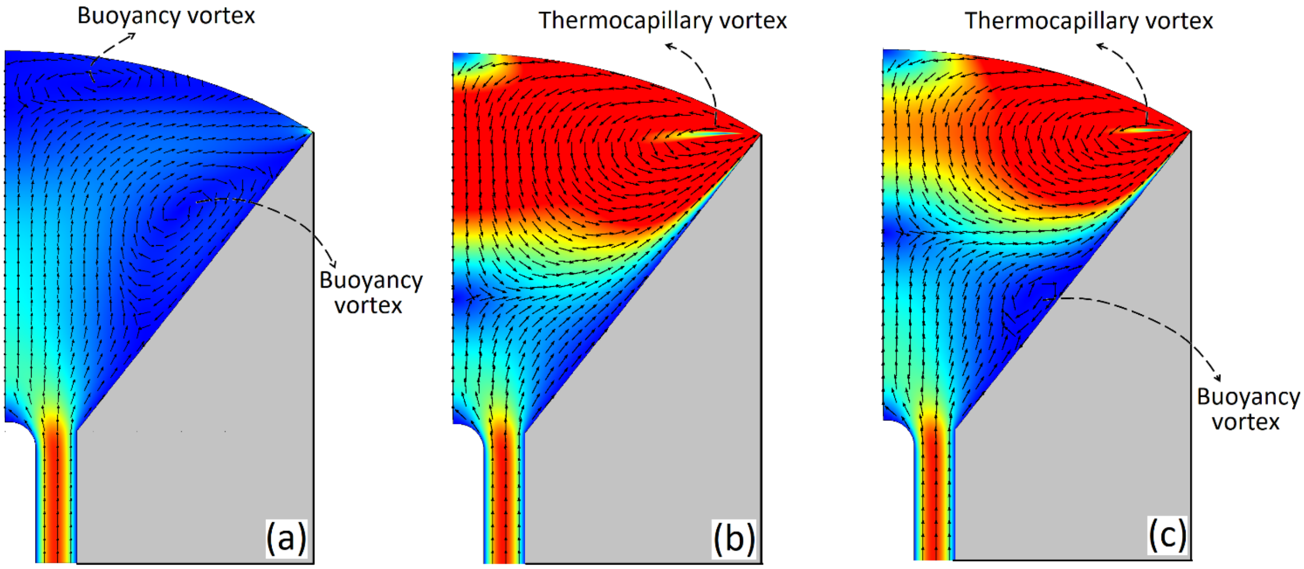

Figure S3. Interaction between the thermocapillary and buoyancy effects for evaporation from an axisymmetric reversed-flow funnel (Ward and Duan[33]) obtained from the simulation. (a) shows the liquid flow in the absence of thermocapillary effects (surface tension set to a constant). (b) shows the liquid flow in the absence of buoyancy effects (gravitational acceleration g set to zero). (c) shows the liquid flow when both effects are activated. The simulation is performed for pressure in the vapor of P^V = 300 Pa and wall thermal conductivity of k_s = 16 W/(m K). The simulated evaporation fluxes for (a), (b), and (c) are 3.85×10^{-3} kg/(m^{2}s), 4.33×10^{-3} kg/(m^{2}s), and 4.26×10^{-3} kg/(m^{2}s), respectively.
